# Investigating the Ultrasonic Pulse Velocity of Concrete Containing Waste Marble Dust and Its Estimation Using Artificial Intelligence

**DOI:** 10.3390/ma15124311

**Published:** 2022-06-17

**Authors:** Dawei Yang, Jiahui Zhao, Salman Ali Suhail, Waqas Ahmad, Paweł Kamiński, Artur Dyczko, Abdelatif Salmi, Abdullah Mohamed

**Affiliations:** 1 Civil & Architecture Engineering, Xi’an Technological University, Xi’an 710021, China; xingqitian@sina.com; 2 Department of Civil Engineering, University of Lahore (UOL), 1-Km Defence Road, near Bhuptian Chowk, Lahore 54000, Pakistan; salmanalisuhail@gmail.com; 3 Department of Civil Engineering, COMSATS University Islamabad, Abbottabad 22060, Pakistan; 4 Faculty of Civil Engineering and Resource Management, AGH University of Science and Technology, 30-059 Krakow, Poland; pkamin@agh.edu.pl; 5 Mineral and Energy Economy Research Institute of the Polish Academy of Sciences, J. Wybickiego 7a, 31-261 Krakow, Poland; arturdyczko@min-pan.krakow.pl; 6 Department of Civil Engineering, College of Engineering, Prince Sattam bin Abdulaziz University, Al-Kharj 16273, Saudi Arabia; a.salmi@psau.edu.sa; 7 Research Centre, Future University in Egypt, New Cairo 11845, Egypt; mohamed.a@fue.edu.eg

**Keywords:** waste, marble dust, building materials, mortar, concrete

## Abstract

Researchers and engineers are presently focusing on efficient waste material utilization in the construction sector to reduce waste. Waste marble dust has been added to concrete to minimize pollution and landfills problems. Therefore, marble dust was utilized in concrete, and its prediction was made via an artificial intelligence approach to give an easier way to scholars for sustainable construction. Various blends of concrete having 40 mixes were made as partial substitutes for waste marble dust. The ultrasonic pulse velocity of waste marble dust concrete (WMDC) was compared to a control mix without marble dust. Additionally, this research used standalone (multiple-layer perceptron neural network) and supervised machine learning methods (Bagging, AdaBoost, and Random Forest) to predict the ultrasonic pulse velocity of waste marble dust concrete. The models’ performances were assessed using R^2^, RMSE, and MAE. Then, the models’ performances were validated using k-fold cross-validation. Furthermore, the effect of raw ingredients and their interactions using SHAP analysis was evaluated. The Random Forest model, with an R^2^ of 0.98, outperforms the MLPNN, Bagging, and AdaBoost models. Compared to all the other models (individual and ensemble), the Random Forest model with greater R^2^ and lower error (RMSE, MAE) has a superior performance. SHAP analysis revealed that marble dust content has a positive and direct influence on and relationship to the ultrasonic pulse velocity of concrete. Using machine learning to forecast concrete properties saves time, resources, and effort for scholars in the engineering sector.

## 1. Introduction

Keeping in mind sustainable development, the need is to curtail excessive industrial processes, along with the enhancement of cost efficiency in parallel with a reduction in environmental pollution [1]. Industrial waste, when incorporated in concrete, can contribute towards sustainable development in terms of environmentally friendly and economical construction materials [2,3]. The partial replacement of cement and other constituents of concrete has already been made extensively by industrial byproducts in various studies [4,5,6,7,8]. Several types of waste materials have been studied for their potential use in building materials, such as marble [9,10,11,12], super-absorbent polymer [13,14], glass [15,16,17], slag [18], bagasse ash [19], rubber [20,21], plastic [22], ceramic [23,24], natural fiber [25,26,27,28], and recycled aggregate [29,30,31,32]. Among these, marble dust, which is produced during cutting processes in mines, has also been used in the production of concrete. The use of marble dust, either as a natural aggregate [33,34,35] or as a replacement for Portland cement (PC) [36,37,38], has been studied in various research. The major focus of existing studies has been replacing cement with alternative sustainable materials to reduce emissions caused by PC. Marble waste has been used as a cement replacement in concrete by various researchers [33,35,39,40,41,42]. However, Li, et al. [43] reported the reduced emissions with 10% marble dust replacement in concrete. Li, et al. [44] and Li, et al. [43] also proposed a paste replacement method for reducing significant (i.e., 33%) cement content and enhancing the utilization of marble dust waste with enhanced durability and strength. Marvila, et al. [10] conducted research on cement and lime mortars using marble waste as a complementary binder. The authors observed that the results were satisfactory, with an increase in mechanical strength with the use of marble waste. However, as a result of technology advancements, laboratory testing is increasingly inadequate and uneconomical due to the time and expense involved.

The mechanical characteristics of concrete can now be predicted using machine learning (ML) methods, owing to advances in artificial intelligence (AI) [45]. Classification, clustering, and regression are examples of machine learning approaches that can be used to estimate a variety of parameters with varying degrees of effectiveness and predict the precise ultrasonic pulse velocity of concrete. As a result of recently evolved artificial intelligence, the mechanical properties of different material types can be forecasted with the help of supervised machine learning (ML) algorithms [46]. ML approaches, e.g., classification, regression, and clustering, are deployed for statistical processes and for the prediction of compressive strength with high accuracy [47]. The accuracy of the prediction can be enhanced by the integration of standalone models, which yields an ensemble machine learning (EML) model, as depicted by other fields of study [48,49]. The employment of ensemble learning for the prediction of concrete parameters has been studied with a limited scope. Random Forest and adaptive boosting (AdaBoost) are EML techniques that can enhance prediction accuracy through the combination of voting and various regression tree forecasting on the ultimate result [50]. Song, et al. [51] determined the compressive strength of ceramic-waste-modified concrete both experimentally and with standalone techniques. The marginal variation in the experimental results and the prediction model outcomes were reported. Accordingly, the current study aims at investigating the usage of advanced techniques for forecasting concrete properties. Ahmad, et al. [50] performed both EML and standalone techniques for the prediction of concrete’s compressive strength and accuracy comparison. It was reported that the outcome predicted by the EML techniques had more accuracy than that of the standalone technique. However, the range of the standalone technique results was also acceptable.

Taking into account the above-mentioned issues, NDT techniques are becoming an emerging alternative solution nowadays. Rebound hammer and ultrasonic pulse velocity (UPV) are the most commonly employed techniques [52,53], both in situ and in the laboratory, as per European standards [54,55]. The quality and homogeneity of different materials such as rocks, wood, and concrete can be evaluated using a nondestructive test named ultrasonic pulse velocity (UPV). In the said test, computation of the velocity using an ultrasonic wave pulse that travels through the considered concrete structure is considered to determine the quality and strength of concrete. The time required for the said pulse to dissipate through the test specimen is measured. The ratio of the test specimen’s width to the time consumed by the wave pulse for dissipation is called pulse velocity. The ultrasonic wave speed relies on Young’s modulus and the density of the testing element. Great care must be given while performing the test, although it is easy to conduct a UPV test. The applicability of a UPV test is in the field, as well as in the laboratory. Both deterioration analysis and quality control can be conducted using UPV. However, higher accuracy can be achieved by considering both values to predict the strength of concrete. Even so, it has been revealed from experimental outcomes that the developed individual machine learning models can achieve predictions with more accuracy. However, ensemble machine learning models are gaining popularity these days; therefore, a performance comparison between these models is necessary. In addition, in the designing phase of projects, it may be an effective alternative for assisting civil engineers.

Only data regarding concrete composite mix proportions are usually accounted for in various studies as input variables, instead of performing other additional measurements. However, knowledge about the combined application of prediction models with NDT techniques is still missing, pointing towards a research gap. Accordingly, the main aim of the current study is to explore a reliable yet simple method for predictions of UPV for waste marble concrete composites. Waste marble dust in concrete is explored in terms of ultrasonic pulse velocity prediction through the application of artificial intelligence, as presented in the current study. Nondestructive testing data are used for this prediction, and its performance with existing artificial intelligence models, considering the effect of raw ingredients and their interactions using SHAP analysis, is claimed to be the novelty of the current research. To tackle challenges such as the excessive consumption of time and money, novel machine learning algorithms are presented for anticipating the behavior of waste concrete in terms of NDT. The focus of this research is to examine the UPV of marble waste concrete and its estimation using an artificial intelligence approach. The current work is unique in that it conducts experiments on waste marble concrete and uses computational models for the prediction of UPV. This study is important for understanding the significance of input parameters and their correctness in ML algorithm results. The findings of the experimental work are also compared to the results of individual ML and ensemble techniques in this study. Each model’s performance is additionally assessed using k-fold cross-validation and statistical tests. Furthermore, a technique [56] is also employed for the attainment of the implemented ML models’ enhanced explanation with the help of global feature influence classification and the respective feature dependencies and interactions. This technique discovers a novel area of knowledge in the form of marble dust concrete ingredients’ influences on UPV, which is beneficial to researchers for classifying suitable design mixes for marble dust concrete and for rapidly forecasting the UPV of marble dust concrete without performing trial and error experimentation. The above-mentioned knowledge area is also helpful for conducting studies in the future for the strategic establishment of marble dust concrete with advanced functional and mechanical features depending upon numerous limitations, such as time, cost, materials, and UPV requirements, for various projects in the construction industry.

## 2. Materials and Methods

**Figure 1:**
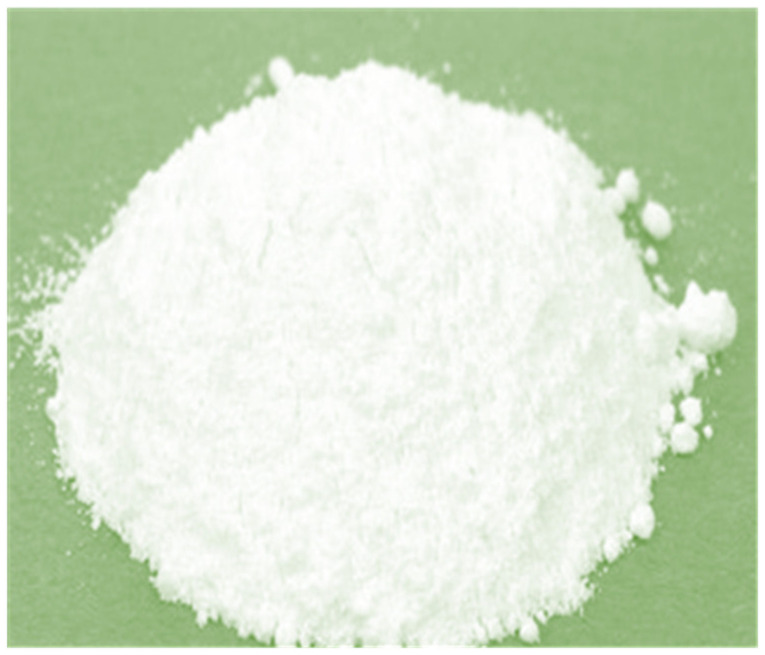
Marble dust.

**Figure 2:**
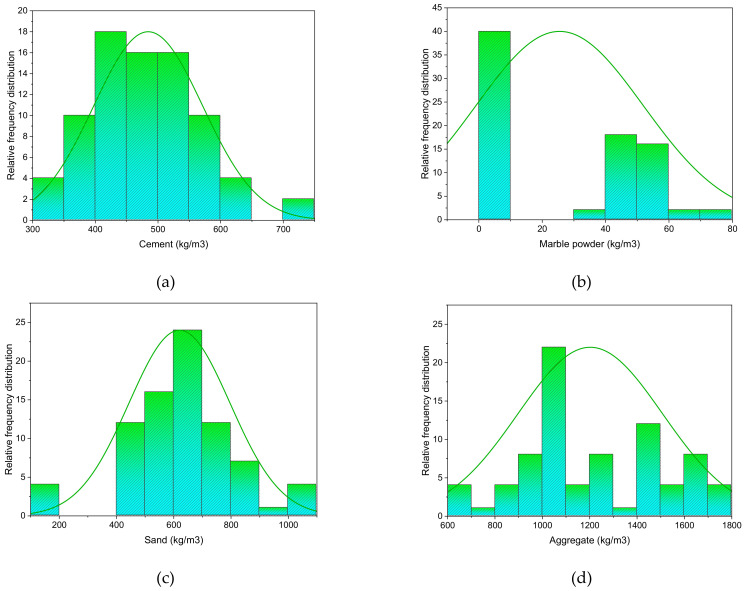
Relative frequency distribution of input parameters: (**a**) cement; (**b**) marble dust; (**c**) sand; and (**d**) coarse aggregate.

**Figure 3:**
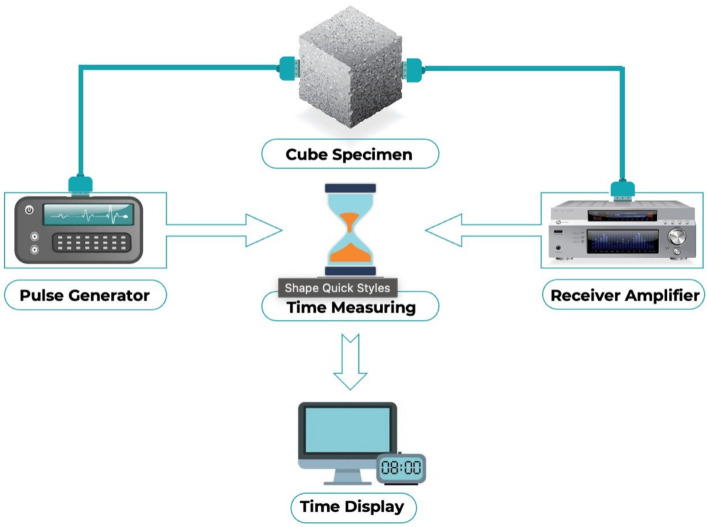
UPV testing procedure.

The raw materials included cement and marble dust, as well as fine and coarse aggregates. For Type I OPC, the Blaine fineness value was 2196 m^2^/kg, and the relative density was 2.43 g/cm^3^. The marble powder had a large specific surface area, which suggests that adding it to concrete would improve its cohesiveness. An XRF technique was performed in order to check the chemical composition. The physical properties were determined using ASTM standards, i.e., ASTM C136, ASTM C29, ASTM C566, and ASTM C128/C127. Table 1 lists the chemical content of the used marble dust, and Figure 1 shows the physical appearance of the marble dust. Silicon dioxide in an amount of 73% was found in the sand sample using an XRF technique. Locally accessible coarse aggregates up to 25.4 mm in nominal size were employed. The Type I cement’s surface area was 385 m^2^/kg. The specific gravities of the sand and aggregate were 2670 and 2650 kg/m^3^, respectively. Detailed information about the properties of the raw materials is available in a previous study [51]. Figure 2 depicts the frequency distribution of each component used in the mixes. It is related to distribution probability, which represents the number of observations linked with a set of values or a single value. Table 2 also shows the physical parameters of the fine and coarse aggregates. This research compares two mix designs, i.e., 20 different mixes for controlled concrete and 20 different mixes for marble-replaced concrete. A marble content of 10% has been suggested in the literature for optimized properties. Therefore, 10% marble waste was used in all the mixes for prediction using the artificial intelligence approach. The study was designed to estimate the UPV using machine learning techniques, and this was the main reason for selecting different types of mixes. Three cube specimens of 150 mm^3^ were prepared for each mix. After demolding, the specimens were water-cured for 28 days. The ASTM C192/C192M was followed for the making and curing of the test specimens of the concrete. Then, ASTM C597 was followed to determine the ultrasonic pulse velocity of the concrete, as shown in Figure 3.

**Figure 4:**
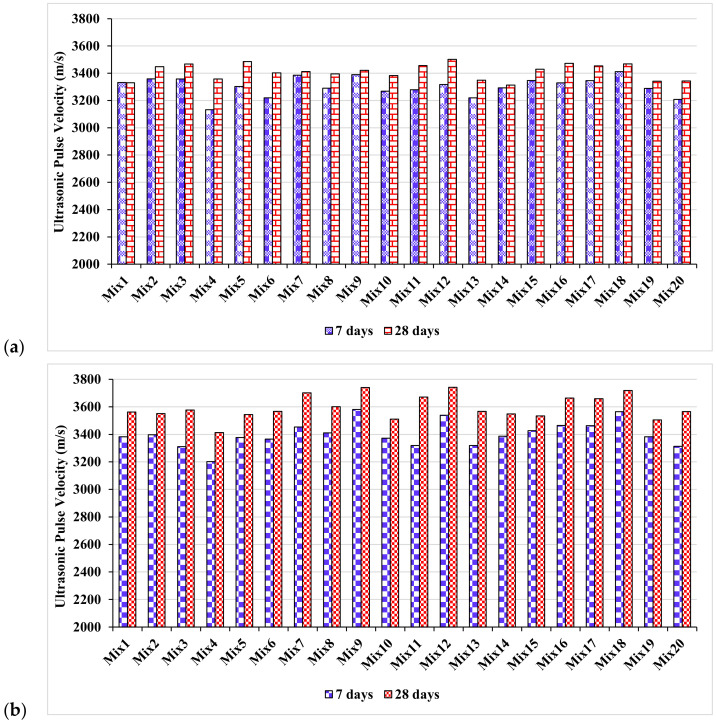
Experimental ultrasonic pulse velocity of mixes: (**a**) control; and (**b**) waste marble dust.

**Figure 5:**
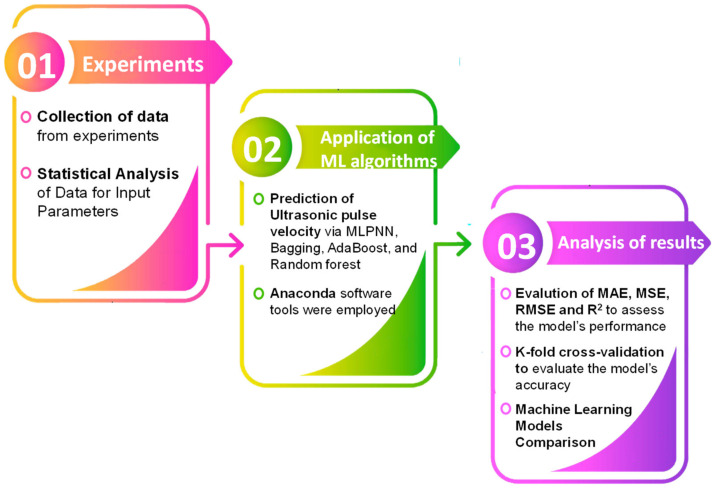
Research methodology with application of machine learning for this study.

The test results showed that an increase in UPV was observed with the addition of marble dust in the concrete. The UPV results of the controlled and waste marble dust mixes are presented in Figure 4a,b, respectively. The UPV of the waste marble dust concrete was higher than that of the controlled concrete. Calcium carbo-aluminate, which is formed in concrete due to a reaction with the CaCO_3_ in marble dust, accelerates both the hydration rate and strength development [37]. A greater pulse velocity indicated homogeneity and excellent quality, whereas a lower pulse velocity indicated nonhomogeneity. The methodology of the current research with the application of machine learning is shown in Figure 5.

The dataset comprised 6 inputs: cement, marble dust, w/c ratio, coarse aggregates, sand, and days. Table 3 describes the statistical analysis of the input parameters. Except for age, which was evaluated in days, all the characteristics were weighted in kg/m^3^. The findings of the descriptive analysis were dependent on many input factors. The table provides the lowest and maximum values and ranges for each variable utilized in the model. Other analytic parameters used to show the relevant values include standard deviation, mean, mode, and a total of all the data points for each variable.

## 3. Results and Discussion

This section addresses the ultrasonic pulse velocity prediction algorithms. A single-layer perceptron neural network (MLPNN) was used as an individual algorithm, while Bagging, AdaBoost, and Random Forest models were implemented as ensemble ML approaches using Python code with Anaconda software. These algorithms are generally used to anticipate outcomes based on input factors. All the techniques used six input parameters and one output parameter (ultrasonic pulse velocity) during the modeling phase. All the ensemble models were shown to be accurate and valid, as discussed below.

### 3.1. Multiple-Layer Perceptron Neural Network (MLPNN) Algorithm

**Figure 6:**
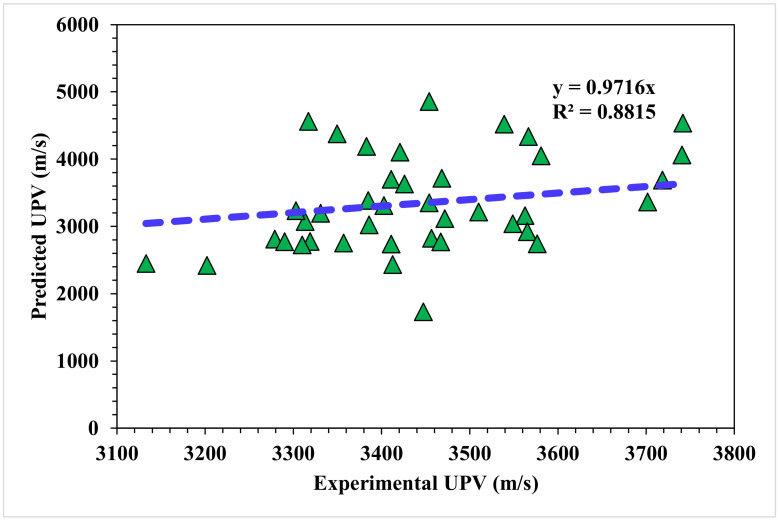
MLPNN model experimental and predicted results.

**Figure 7:**
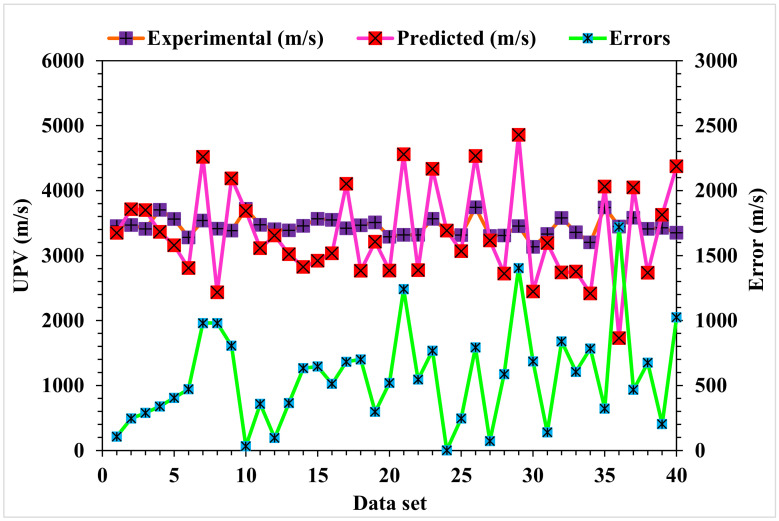
MLPNN model experimental and predicted values with the errors.

Figure 6 depicts the statistical analysis of the predicted and actual results regarding the UPV of WMDC for MLPNN modeling. A reasonably précised output and very low variation between the anticipated and actual values was obtained with the MLPNN technique. The accuracy of predicting results was assessed as having a 0.88 R^2^ value. The dispersions for the predicted and experimental values (targets) with the MLPNN model errors are shown in Figure 7. The average, highest, and lowest values of the training set were 6.20, 20.7, and 0.07 MPa, respectively. A total of 45% of the error values were less than 500 m/s, 45% were from 500 to 1000 m/s, and 10% were higher than 1000 m/s.

### 3.2. Bagging Algorithm

**Figure 8:**
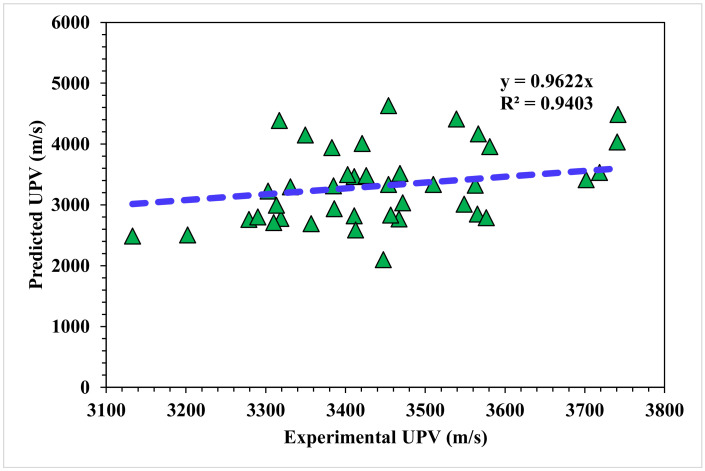
Bagging model experimental and predicted results.

**Figure 9:**
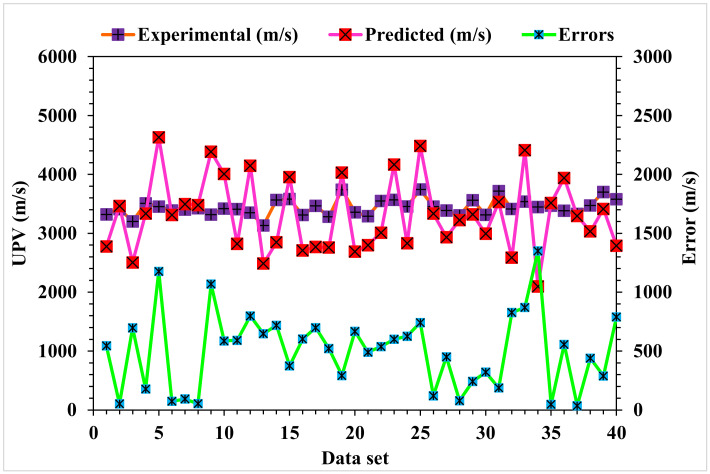
Bagging model experimental and predicted values with the errors.

The correlation between the projected and actual results of the Bagging model is shown in Figure 8. The R^2^ value for the Bagging model was 0.94, which represents the highly precise and more accurate Bagging model with respect to the MLPNN model. Furthermore, the dispersion of the projected values, the actual targeted values, and the errors for the Bagging model are shown in Figure 9. It was noted that 45% of the error data was below 500 m/s, 47.5% was from 500 to 1000 m/s, and only 7.5% was higher than 1000 m/s. The higher accuracy of the Bagging model with respect to the MLPNN model was revealed from this analysis. It was also depicted by lower error and greater R^2^ values. In addition, twenty submodels were employed using EML methods (MLPNN, AdaBoost, and Random Forest) to obtain an optimized value that produced a firm output.

### 3.3. AdaBoost Algorithm

**Figure 10:**
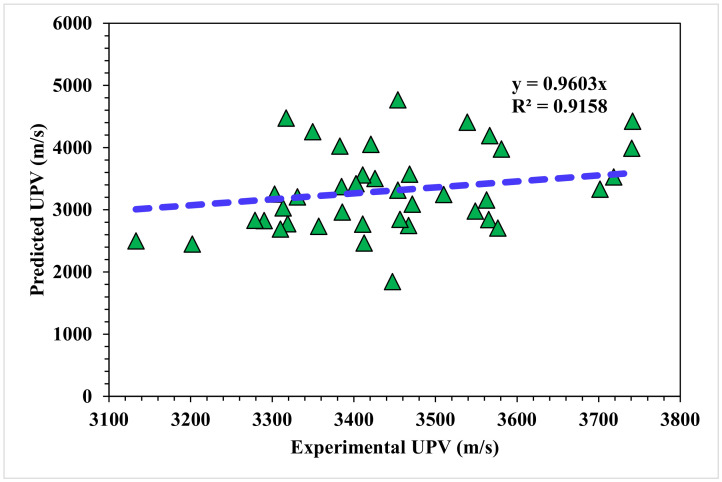
AdaBoost model experimental and predicted results.

**Figure 11:**
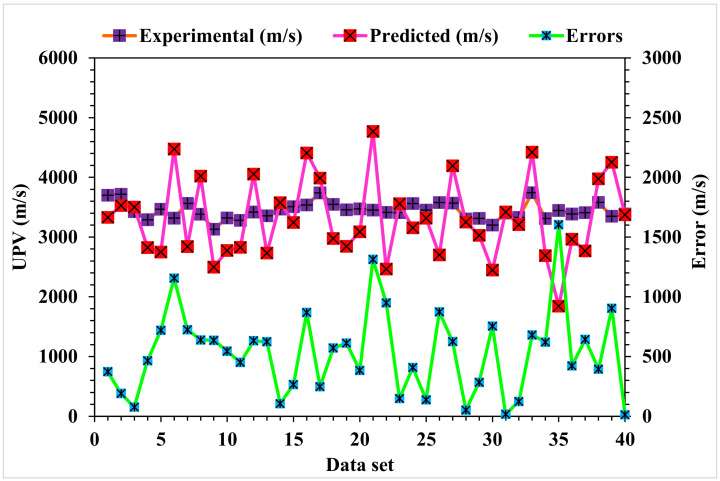
AdaBoost model experimental and predicted values with the errors.

A comparison of the projected and actual outputs for the AdaBoost model is shown in Figure 10. The R^2^ value was 0.91, which showed a better outcome when compared to the MLPNN model. The dispersions of the actual and predicted values with the errors for the AdaBoost model are illustrated in Figure 11. However, 47.5% of the error values were below 500 m/s, 45% ranged from 500 to 1000 m/s, and only 7.5% were higher than 1000 m/s. The higher accuracy of the AdaBoost model in comparison with the MLPNN model was also depicted by lower error values.

### 3.4. Random Forest Algorithm

**Figure 12:**
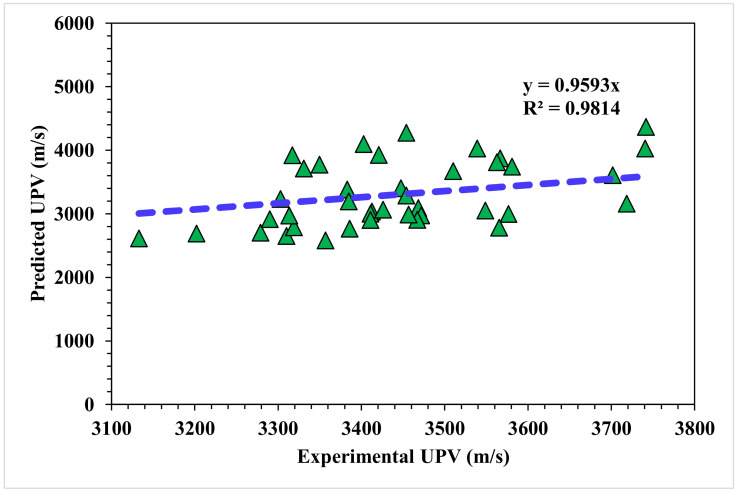
Random Forest model experimental and predicted results.

**Figure 13:**
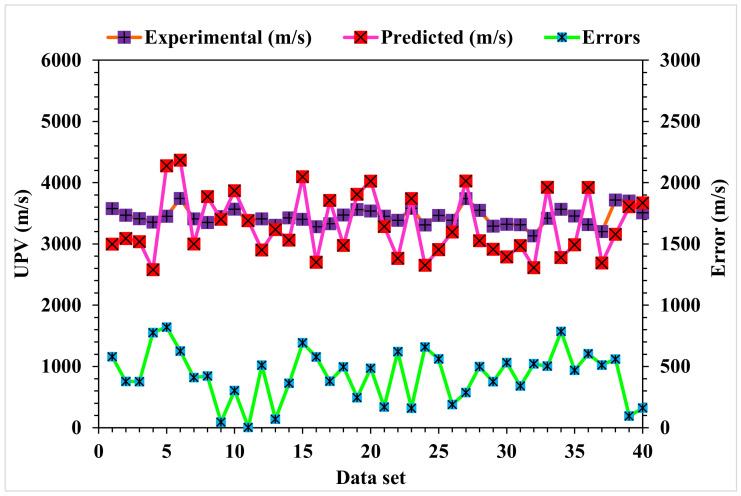
Random Forest model experimental and predicted values with the errors.

The correlation between the predicted and actual output values for the Random Forest model is provided in Figure 12. The R^2^ value for this model came out to be 0.98, showing considerable accuracy compared to the MLPNN, Bagging, and AdaBoost models. The dispersions of the actual and predicted values with the errors for the Random Forest model are shown in Figure 13. Only 57.5% of the error values were below 500 m/s, 42.5% of the values ranged from 500 to 900 m/s, and no values were found above 900 m/s. The error distribution and R^2^ values were more accurate than the MLPNN, Bagging, and AdaBoost models for the UPV prediction of WMDC. The R^2^ values, along with the error values, obtained from all the considered ensemble ML models were in an acceptable range, depicting better prediction outcomes. Hence, it was observed in this study that EML techniques (Random Forest, followed by Bagging and Adaboost) predicted high-accuracy outcomes when compared to a standalone MLPNN technique.

## 4. Model Performance Assessment

### 4.1. K-Fold Cross-Validation Checks

**Figure 14:**
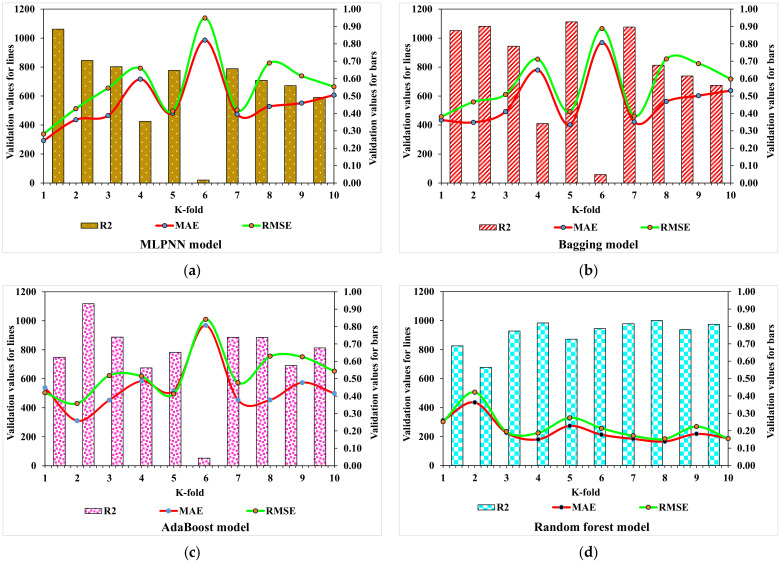
K-fold cross-validation: (**a**) MLPNN model; (**b**) Bagging model; (**c**) AdaBoost model; and (**d**) Random Forest model.

Statistical analyses with Equations (1) and (2) were utilized to predict the responses of the models. The legitimacy of the models was evaluated by utilizing a k-fold cross-validation approach during execution. Usually, the validity of a model is performed with a k-fold cross-validation process [57] in which random dispersion is perfomed by splitting the model into 10 groups. The greater the R^2^ value and the fewer the errors (RMSE and MAE), the higher the accuracy of the model. Furthermore, this process should be repeated multiple (i.e., 10) times for a satisfactory result. The exceptional precision of a model can be achieved by using this comprehensive approach. In addition, statistical analyses (i.e., RMSE and MSE) were also performed for all the models (Table 4). The Random Forest model accuracy (inversely related to error values) compared to the AdaBoost, Bagging, and MLPNN models was also supported by these checks. Statistical analysis as reported in the literature [47,58] is used to assess the response of a model to prediction. The k-fold cross-validation is assessed by utilizing R^2^, RMSE, and MAE. Respective dispersions for the DT, Random Forest, AdaBoost, and Bagging models are presented in Figure 14. The average and maximum values of R^2^ for the MLPNN were 0.55 and 0.88, respectively (refer to Figure 14a). The maximum and average values of R^2^ for the Bagging model were 0.94 and 0.66, respectively, as shown in Figure 14b. Contrary to this, the maximum and average R^2^ values of the AdaBoost model were 0.91 and 0.62, respectively, as portrayed in Figure 14c. In comparison, the maximum and average values of R^2^ for Random Forest were 0.98 and 0.76, respectively (see Figure 14d). To compare the error values (RMSE and MAE), the RMSE and MAE values for all the models are shown in Table 4. The Random Forest model, with the lowest error and a higher R^2^ value, performed better in results prediction.

(1) $$\mathrm{MAE}=\frac{1}{n}\overset{n}{\underset{i=1}{\sum }}\left|x_{i}-x\right|$$

(2) $$\mathrm{RMSE}=\sqrt{\sum \frac{{\left(y_{pred}-y_{ref}\right)}^{2}}{N}}$$

where *n* is the number of total data samples, x and $y_{ref}$ are the data sample reference values, and $x_{i}$ and $y_{pred}$ are the model prediction values.

### 4.2. Comparison of Machine Learning Models

**Figure 15:**
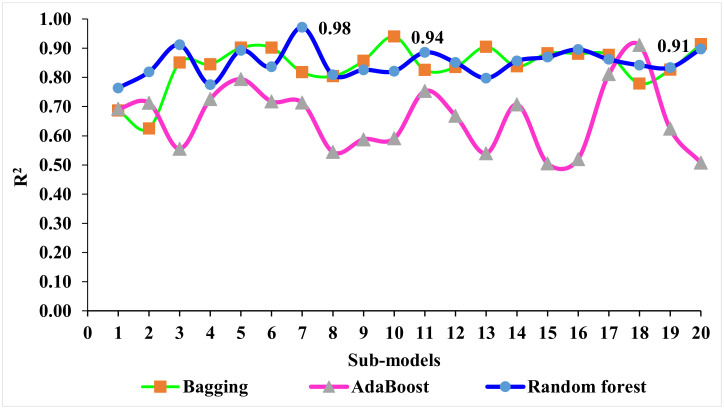
R^2^ values of submodels.

Both ensemble ML and individual approaches were explored in this study for the estimation of WMDC with the aim of sustainable development in terms of environment-friendly construction materials. Random Forest, Bagging, AdaBoost, and MLPNN machine learning techniques were used in this study to predict the compressive strength of WMDC. The goal of the MLPNN algorithm was the development of a model that could predict the target variable accurately. On the other hand, for the Bagging technique, a random sample was selected from the data of the training set, i.e., the selection of individual data points could be made multiple times. The individual training of the said weak models was conducted in the pursuance of numerous data sample generation and based on task type, such as classification or regression or average or majority of these predictions to give an estimate with high accuracy. For the establishment of an algorithm’s prediction superiority, the employed algorithms were compared for targeted performance. MLPNN and Random Forest are two alternative learning techniques that can be utilized in similar applications. The main rationale for using a Random Forest rather than an individual decision tree or MLPNN was that it allowed the aggregation of predictions of multiple decision trees in a single model. The theory was that a single model comprised of numerous poor models is still preferable to a single good model. Given the widespread performance of Random Forests, this s true. As a result, Random Forests are less prone to overfitting. Random Forest’s major benefit is that it relies on a collection of different decision trees to arrive at any solution. It is an ensemble method that takes into account the findings of multiple classifying algorithms of the same or different types. It is capable of both regression and classification. A Random Forest generates accurate predictions that are simple to comprehend. It is capable of effectively handling huge datasets. In comparison to the individual MLPNN method, the Random Forest algorithm is more accurate at predicting outcomes. The sklearn (Scikit-learn) library was used, and 50% of the data were taken for training purposes and 50% for testing. The output of the Random Forest model was more accurate, having a 0.98 R^2^ value, in comparison to Bagging with 0.94 R^2^, AdaBoost with 0.91 R^2^, and MLPNN with 0.88 R^2^. Furthermore, the performances of the MLPNN, Bagging, AdaBoost, and Random Forest models were also evaluated by utilizing a k-fold cross-validation technique and statistical analysis. The performance of the model was higher with low error levels. However, it was difficult to assess optimized machine learning regressors to forecast results from a wide range of topics because the performance of the model was very much dependable on the datapoints and the model’s input parameters. On the other hand, for ensemble ML techniques, submodels were generated to leverage the weak learner that could be optimized and trained with data for achieving a higher value of R^2^. Other researchers have also observed that AdaBoost, Bagging, and RF models are more accurate in predicting outcomes than individual machine learning techniques [45,50,59,60,61]. Feng, et al. [45] observed that an AdaBoost model outperformed individual models, including an artificial neural network (ANN) and a support vector machine (SVM), in terms of R^2^ and error values. In addition, Ahmad, et al. [50] compared the performances of Bagging, AdaBoost, gene expression programming (GEP), and DT and concluded the best predictor was the Bagging algorithm, with an R^2^ of 0.92. Similarly, Farooq, et al. [60] compared the performance of Random Forest with those of ANN, GEP, and DT approaches and found that the Random Forest model had greater precision than the others, with an R^2^ of 0.96. A higher accuracy for Random Forest was also reported in the literature, having an R^2^ of 0.98 to calibrate a low-cost particle monitor. The dispersion of values for the determinant coefficient of the Bagging, AdaBoost, and Random Forest submodels is shown in Figure 15. The values of R^2^ for all the submodels of Random Forest were greater than 0.76, as shown in Figure 15, while most values of R^2^ in the cases of the submodels for AdaBoost and Bagging were less than 0.63 and 0.51 (Figure 15), respectively. It depicts the higher accuracy of the Random Forest technique for results prediction, showing a maximum value of R^2^, i.e., 0.98. Therefore, the Random Forest model was suggested to predict the ultrasonic pulse velocity of waste marble dust concrete.

### 4.3. Effect of Raw Ingredients and Their Interactions Using SHAP Analysis

An in-depth ML model explanation was made in the current research. In addition to this, the respective feature dependencies and interactions were also discovered. Initially, the implementation of a SHAP tree explainer for the entire dataset was performed for the provision of an enhanced global feature impact description by the mergence of SHAP descriptions. A tree explainer, i.e., a tree-like SHAP approximation technique, was employed [62]. In this technique, the tree-based model’s internal structure, i.e., the sum of the calculation set linked with a leaf node of the tree model that leads to low-order complexity, is assessed [62]. The highest-precision prediction model was obtained by the Random Forest algorithm for the UPV of marble dust concrete. Accordingly, the model interpretation was made for the UPV of marble dust concrete with the help of SHAP analysis.

**Figure 16:**
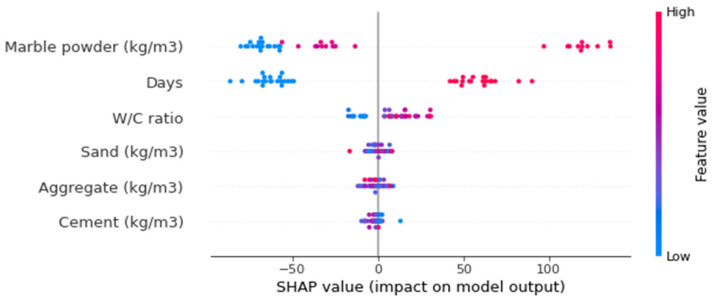
SHAP plot.

Figure 16 depicts the violin SHAP-plot values of the considered features for the prediction of UPV for marble dust concrete. A unique color is used to show the feature values in this plot, and the *x*-axis-corresponding SHAP value represents the output contribution. For example, for marble dust, the content input feature had a higher impact and positive influence, showing the direct relation of this feature with the UPV of marble dust concrete. This means that an increasing content of marble would result in a higher UPV value. A SHAP value of more than 100 in the form of red points (high-value color) at the rightmost side depicts that higher marble dust content enhanced the marble dust concrete UPV. In the case of the curing age feature, a positive influence was seen here as well. At 7 days of age, it is depicted in blue, showing a lower value. Whereas, at 28 days, it increased, as depicted from the higher, i.e., red, values on the right side of the axis. However, in the case of the water content feature, both positive and negative influences are depicted. The water content up to the optimum content was influenced positively; beyond that, there was a negative influence on the UPV of marble dust concrete. In the case of considerably decreased water content, it was also negatively influenced due to affected compaction, resulting in enhanced porosity and, ultimately, a decreased UPV of marble dust concrete. Similarly, sand, aggregate, and cement had more or less the same influence and were on the border of having both positive and negative influences. This evaluation relied on the dataset employed in this study, and high-precision outcomes may also be achieved with more datapoints.

**Figure 17:**
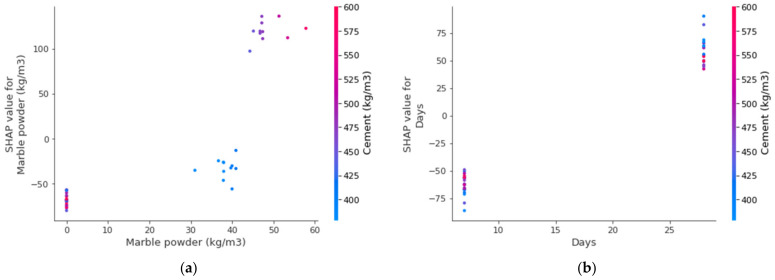

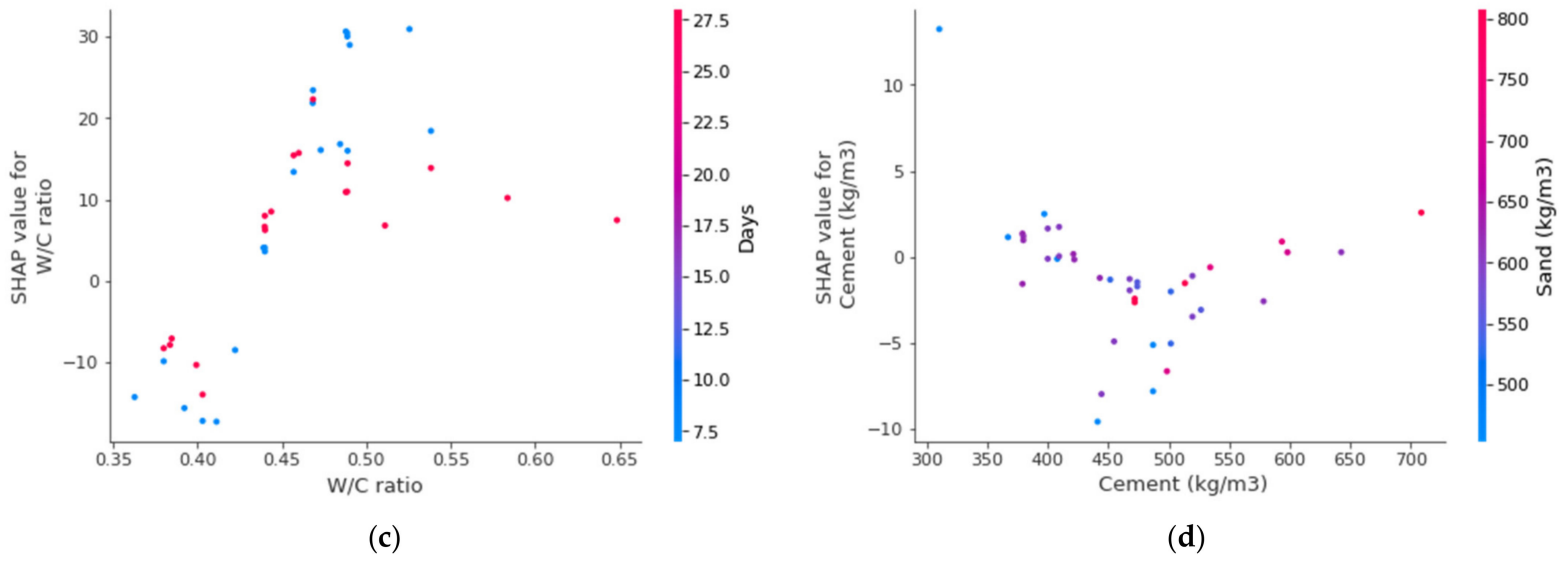
Interaction plots of various parameters: (**a**) marble dust; (**b**) days; (**c**) w/c ratio; and (**d**) cement.

The feature interactions with the UPV of marble dust concrete are presented in Figure 17. The marble dust feature interaction is shown in Figure 17a. It can be observed from the plot that marble dust positively interacted with the UPV of marble dust concrete and was in a positive–direct relationship. It may also be noted that, among all the features, marble dust majorly interacted with cement, as it was used as a cement replacement. In Figure 17b, the positive influence of curing days on the UPV of marble dust concrete is observed because more interaction of days with the cement hydration process ultimately increased the strength and UPV of the concrete. The w/c feature interaction is plotted in Figure 17c. The w/c indicated both negative and positive impacts, depending upon its content. The major interaction of w/c was with the cement content, as both water and cement have a link to the hydration process, which is mainly dependent on curing age (days). Then, the cement content feature interaction with sand did not show any particular trend (Figure 17d) and showed almost the same pattern.

Although SHAP was used for the interpretations in this study, there are numerous other post hoc explanatory models that can be used for the same purpose. As a result, we recommend comparing the interpretations obtained using various explanation methodologies. The SHAP-plot values estimated using SHAP, for example, may differ from those obtained using other explanation approaches. Furthermore, the research focused on concrete’s UPV. The study, however, can be applied to other strength parameters as well, such as compressive strength, etc. Other strength features need to be predicted using ML in conjunction with post hoc explainable approaches, and the underlying rationales are required to be explained. As a result, the influencing parameters that are required for the design stage can be discovered using this approach, but they still need to be investigated in the future.

## 5. Conclusions

The incorporation of marble waste dust into concrete can be an efficient way to improve the environment and reduce landfill pollution. To achieve this, waste marble dust was used in concrete. Additionally, soft computing techniques were compared to predict waste marble dust concrete (WMDC) characteristics. Based on the conducted research, the following conclusions were drawn:

- An amount of 10% marble dust in concrete influenced the ultrasonic pulse velocity. The ultrasonic pulse velocity increased due to the reduced porosity of concrete with marble dust. In this case, waste marble dust concrete with 10% marble dust (as a replacement) showed improved UPV compared to the control mix with 0% marble dust.
- Due to its greater R^2^ and lower error levels, the Random Forest model outperformed AdaBoost, Bagging, and MLPNN techniques in terms of prediction. The MLPNN, Bagging, AdaBoost, and Random Forest models had R^2^ values of 0.88, 0.94, 0.91, and 0.97, respectively. However, the ensemble model results for Random Forest, followed by Bagging and AdaBoost, were acceptable.
- A k-fold cross-validation technique and statistical analyses revealed adequate Random Forest, AdaBoost, and Bagging outcomes. These tests also showed that the Random Forest model outperformed the MLPNN, AdaBoost, and Bagging models.
- The study validated the application of ultrasonic pulse velocity for forecasting the ultrasonic pulse velocity of sustainable cementitious composite. Therefore, the presented techniques using artificial intelligence seemed reliable for predicting waste marble dust concrete properties.
- A higher SHAP-plot value depicted the positive relation of marble dust content with the UPV of marble dust concrete.
- The feature interaction plot represented that marble dust and curing days positively interacted with cement content and improved the UPV of concrete.

This study was limited to the prediction of the UPV of waste marble dust concrete with limited input parameters and machine learning algorithms (an MLPNN-based approach and decision-tree-based approaches). It is suggested that more comprehensive research on waste marble dust needs to be conducted with more criteria included. Adding additional input factors and expanding the database can produce more trustworthy findings and provide a more comprehensive expression. These parameters should include, in the future, compressive strength, temperature effect, acid attack resistance, chlorine resistance, sulphate resistance, and corrosion. Advanced technologies such as particle swarm optimization (PSO) and M5P trees can be used to make more accurate predictions. However, for better results, machine learning approaches can be coupled with heuristic methods, such as the whale optimization algorithm and ant colony optimization, and then compared with the current study. Further studies should be carried out to investigate the chemical properties of waste marble dust, as well as all other mechanical properties that are key to any application in concrete.

## Figures and Tables

**Table 1:** Chemical composition.

Components	Marble Dust	Cement
SiO_2_	14.08	18.93
Al_2_O_3_	2.69	9.89
MgO	2.77	1.67
CaO	42.14	59.6
K_2_O	0.63	1.13
Na_2_O	0.61	0.90
Fe_2_O_3_	1.94	3.59

**Table 2:** Physical properties of raw materials.

Parameters	Maximum Size	Fineness Modulus	Moisture Content	Density
	mm	-	%	kg/m^3^
Cement	-	-	-	1432
Marble dust	-	1.86	-	1118
Sand	-	2.72	1.57	1790
Coarse aggregate	25.4	-	1.49	1591

**Table 3:** Details of input data.

	Input Data						
	Cement (kg/m^3^)	Marble Dust (kg/m^3^)	Sand (kg/m^3^)	Aggregate (kg/m^3^)	Water (kg/m^3^)	Days	UPV (m/s)
Standard Error	9.73	2.95	19.55	33.62	4.55	1.18	42.34
Median	472.84	17.24	615.26	1116.36	220.83	17.50	3334
Minimum	310.15	0.00	129.47	659.33	130.97	7.00	3110
Maximum	708.80	70.89	1020.65	1750.97	303.96	28.00	4502
Mode	486.95	0.00	620.06	1201.29	185.03	7.00	3357
Mean	484.40	25.49	618.70	1202.28	217.13	17.50	3518
Standard Deviation	86.98	26.39	174.90	300.70	40.67	10.57	378.68
Range	398.65	70.89	891.17	1091.64	172.99	21.00	1392

**Table 4:** Statistical descriptions of MLPNN, Bagging, AdaBoost, and Random Forest models.

Models	MAE (m/s)	RMSE (m/s)	R^2^
MLPNN	564.4	676.7	0.88
Bagging	500.8	594.7	0.94
AdaBoost	531.4	637.6	0.91
Random Forest	429.3	475.7	0.98

## Data Availability

Data available on request from the corresponding authors.
